# Correlation Between Tumor Molecular Markers and Perioperative Epilepsy in Patients With Glioma: A Systematic Review and Meta-Analysis

**DOI:** 10.3389/fneur.2021.692751

**Published:** 2021-09-01

**Authors:** Li Song, Xingyun Quan, Chaoyi Chen, Ligang Chen, Jie Zhou

**Affiliations:** ^1^Department of Neurosurgery, Northwest Women's and Children's Hospital, Xi'an, China; ^2^Department of Neurosurgery, Xi'an International Medical Center, Xi'an, China; ^3^Anorectal Department, Affiliated Hospital of Traditional Chinese Medicine of Southwest Medical University, Lu Zhou, China; ^4^Department of Neurosurgery, The Affiliated Hospital of Southwest Medical University, Lu Zhou, China; ^5^Sichuan Clinical Research Center for Neurosurgery, Lu Zhou, China; ^6^Academician (Expert) Workstation of Sichuan Province, Lu Zhou, China; ^7^Neurological Diseases and Brain Function Laboratory, Lu Zhou, China

**Keywords:** tumor molecular markers, epilepsy, meta-analysis, glioma, perioperative

## Abstract

**Purpose:** Tumors derived from the neuroepithelium are collectively termed gliomas and are the most common malignant primary brain tumor. Epilepsy is a common clinical symptom in patients with glioma, which can impair neurocognitive function and quality of life. Currently, the pathogenesis of glioma-related epilepsy is not fully described. Therefore, it is necessary to further understand the mechanism of seizures in patients with glioma. In this study, a comprehensive meta-analysis was conducted to investigate the relationship between five commonly used tumor molecular markers and the incidence of perioperative epilepsy in patients with glioma.

**Methods:** PubMed, EMBASE, and Cochrane Library databases were searched for related research studies. Odds ratio and the corresponding 95% confidence interval were used as the main indicators to evaluate the correlation between tumor molecular markers and the incidence of perioperative epilepsy in patients with glioma.

**Results:** A total of 12 studies were included in this meta-analysis. The results showed that isocitrate dehydrogenase 1 (IDH1) mutation was significantly correlated with the incidence of perioperative epilepsy. A subgroup analysis showed that IDH1 was significantly correlated with the incidence of preoperative epilepsy, but not with intraoperative and postoperative epilepsy. There was no correlation between O6-methylguanine-DNA methyltransferase methylation and 1p/19q deletion and the incidence of perioperative epilepsy. Tumor protein p53 and epidermal growth factor receptor could not be analyzed because of the limited availability of relevant literature. There was no significant heterogeneity or publication bias observed among the included studies.

**Conclusion:** The present meta-analysis confirms the relationship between tumor molecular markers and the incidence of perioperative epilepsy in patients with glioma. The present results provide more comprehensive evidence for the study of the pathogenesis of glioma-related epilepsy. Our research may offer a new method for the treatment of perioperative seizures in patients with glioma.

## Introduction

Gliomas are the most common malignant tumors of the central nervous system. Seizures occur in 40–70% of patients with glioma (1). Some patients experience epilepsy as the initial symptom, leading to the diagnosis of glioma. Other patients will experience epilepsy during or after tumor resection. Epilepsy reduces the quality of life of patients with glioma and causes huge economic and emotional burdens (2). Despite the availability of antiepileptic drug treatment, approximately half of the patients will develop drug resistance; some patients also continue to experience refractory epilepsy (3). Therefore, it is necessary to systematically investigate the pathogenesis of glioma-related epilepsy.

Previous studies have shown that tumor growth stimulates seizures and *vice versa*, indicating a potential interconnection between the mechanisms of these two pathological conditions (4). A large number of studies have shown that glioma-associated epilepsy is associated with tumor grade and histopathology (4, 5). However, recent studies have shown that tumor molecular markers (TMMs), such as O6-methylguanine-DNA methyltransferase (MGMT) gene promoter methylation, 1p/19q co-deletion, and isocitrate dehydrogenase 1 mutant (IDH1^*mut*^), also affect the occurrence of epilepsy. Nonetheless, due to the small sample size of most studies, the currently available results are inconsistent.

Studies conducted by Stockhammer et al. and Liang et al. showed that preoperative seizures are significantly related to IDH1^*mut*^ (6, 7). However, Mullican et al. concluded the opposite (8). Yang et al. reported that patients with a low expression of the MGMT protein were linked to a higher frequency of postoperative seizures (9). In addition, Feyissa et al. showed that patients with methylation of the MGMT gene promoter were more likely to suffer from postoperative epilepsy (10). Previous studies have shown that 1p/19q co-deletion is related to epileptic seizures in adults (11, 12). Interestingly, Mulligan showed that the existence of 1p/19q co-deletion was not associated with the occurrence of preoperative seizures in patients with low-grade glioma (8). Considering these results, a systematic and comprehensive meta-analysis is required to reach a definitive conclusion. In a meta-analysis conducted in 2018, IDH1 mutations have been associated with a higher incidence of preoperative epilepsy in patients with low-grade glioma (13). However, a comprehensive meta-analysis of other genetic TMMs and glioma epilepsy is lacking. Therefore, the objective of the present study was to examine the relationship between five routinely tested TMMs and perioperative epilepsy in patients with glioma.

## Methods

### Search Strategy

The PubMed, Embase, and Cochrane Library databases were searched from the establishment of each database to May 2020. The search terms used were “epilepsy”, “glioma”, “isocitrate dehydrogenase” or “IDH”, “methyltransferase” or “MGMT”, “ATRX”, “1p/19q”, “P53”, and “EGFR”. The search strategy is shown in detail in Table 1.

**Table 1:** Retrieval strategy.

	**Search**	**Query**
PubMed	#1	((((((((“Isocitrate Dehydrogenase”[Mesh]) OR (Isocitrate Dehydrogenase-I[Title/Abstract])) OR (Isocitrate Dehydrogenase I[Title/Abstract])) OR (IDH[Title/Abstract])) OR ((“Methyltransferases”[Mesh]) OR (MGMT))) OR (ATRX[Title/Abstract])) OR (1p/19q[Title/Abstract])) OR (P53[Title/Abstract])) OR (EGFR[Title/Abstract])
	#2	(((((((((“Glioma”[Mesh]) OR (Gliomas[Title/Abstract])) OR (Glial Cell Tumors[Title/Abstract])) OR (Glial Cell Tumor[Title/Abstract])) OR (Mixed Glioma[Title/Abstract])) OR (Mixed Gliomas[Title/Abstract])) OR (Malignant Glioma[Title/Abstract])) OR (Malignant Gliomas[Title/Abstract])) OR ((((((“Astrocytoma”[Mesh]) OR (Astrocytomas[Title/Abstract])) OR (Astroglioma[Title/Abstract])) OR (Astrogliomas[Title/Abstract])) OR (Astrocytic Glioma[Title/Abstract])) OR (Astrocytic Gliomas[Title/Abstract]))) OR (((((((((“Oligodendroglioma”[Mesh]) OR (Oligodendrogliomas[Title/Abstract])) OR (Mixed Oligodendroglioma-Astrocytoma[Title/Abstract])) OR (Mixed Oligodendroglioma Astrocytoma[Title/Abstract])) OR (Mixed Oligodendroglioma-Astrocytomas[Title/Abstract])) OR (Anaplastic Oligodendroglioma[Title/Abstract])) OR (Anaplastic Oligodendrogliomas[Title/Abstract])) OR (Oligodendroblastoma[Title/Abstract])) OR (Oligodendroblastomas[Title/Abstract]))
	#3	((((((((“Seizures”[Mesh]) OR (Seizure[Title/Abstract])) OR (Complex Partial Seizure[Title/Abstract])) OR (Complex Partial Seizures[Title/Abstract])) OR (Tonic-Clonic Seizures[Title/Abstract])) OR (Tonic Clonic Seizures[Title/Abstract])) OR (Tonic-Clonic Seizure[Title/Abstract])) OR (Clonic Seizures[Title/Abstract])) OR (((((((((“Epilepsy”[Mesh]) OR (Epilepsies[Title/Abstract])) OR (Seizure Disorder[Title/Abstract])) OR (Seizure Disorders[Title/Abstract])) OR (Awakening Epilepsy[Title/Abstract])) OR (Cryptogenic Epilepsies[Title/Abstract])) OR (Cryptogenic Epilepsy[Title/Abstract])) OR (Aura[Title/Abstract])) OR (Auras[Title/Abstract]))
Embase	#1	“isocitrate dehydrogenase”:ab,ti OR “isocitrate dehydrogenase-I”:ab,ti OR “isocitrate dehydrogenase I”:ab,ti OR idh:ab,ti OR methyltransferases:ab,ti OR mgmt:ab,ti OR atrx:ab,ti OR “1p/19q”:ab,ti OR p53:ab,ti OR egfr:ab,ti
	#2	glioma:ab,ti OR gliomas:ab,ti OR “glial cell tumors”:ab,ti OR “glial cell tumor”:ab,ti OR “mixed glioma”:ab,ti OR “mixed gliomas”:ab,ti OR “malignant glioma”:ab,ti OR “malignant gliomas”:ab,ti OR astrocytoma:ab,ti OR astrocytomas:ab,ti OR astroglioma:ab,ti OR astrogliomas:ab,ti OR “astrocytic glioma”:ab,ti OR “astrocytic gliomas”:ab,ti OR oligodendroglioma:ab,ti OR oligodendrogliomas:ab,ti OR “mixed oligodendroglioma-astrocytoma”:ab,ti OR “mixed oligodendroglioma astrocytoma”:ab,ti OR “mixed oligodendroglioma-astrocytomas”:ab,ti OR “anaplastic oligodendroglioma”:ab,ti OR “anaplastic oligodendrogliomas”:ab,ti OR oligodendroblastoma:ab,ti OR oligodendroblastomas:ab,ti
	#3	seizures:ab,ti OR seizure:ab,ti OR “complex partial seizure”:ab,ti OR “complex partial seizures”:ab,ti OR “tonic-clonic seizures”:ab,ti OR “tonic clonic seizures”:ab,ti OR “tonic-clonic seizure”:ab,ti OR “clonic seizures”:ab,ti OR epilepsy:ab,ti OR epilepsies:ab,ti OR “seizure disorder”:ab,ti OR “seizure disorders”:ab,ti OR “awakening epilepsy”:ab,ti OR “cryptogenic epilepsies”:ab,ti OR “cryptogenic epilepsy”:ab,ti OR aura:ab,ti OR auras:ab,ti
Cochrane	#1	(isocitrate dehydrogenase):ti,ab,kw OR (isocitrate dehydrogenase-i):ti,ab,kw OR (isocitrate dehydrogenase):ti,ab,kw OR (idh):ti,ab,kw OR (methyltransferases):ti,ab,kw OR (MGMT):ti,ab,kw OR (atrx):ti,ab,kw OR (“1p/19q”):ti,ab,kw OR (p53):ti,ab,kw OR (egfr):ti,ab,kw
	#2	(glioma):ti,ab,kw OR (gliomas):ti,ab,kw OR (glial cell tumors):ti,ab,kw OR (glial cell tumor):ti,ab,kw OR (mixed glioma):ti,ab,kw OR (mixed gliomas):ti,ab,kw OR (malignant glioma):ti,ab,kw OR (malignant gliomas):ti,ab,kw OR (astrocytoma):ti,ab,kw OR (astrocytomas):ti,ab,kw OR (mixed gliomas):ti,ab,kw OR (malignant glioma):ti,ab,kw OR (malignant gliomas):ti,ab,kw OR (astrocytoma):ti,ab,kw OR (astrocytomas):ti,ab,kw OR (astroglioma):ti,ab,kw OR (astrogliomas):ti,ab,kw OR (astrocytic glioma):ti,ab,kw OR (astrocytic gliomas):ti,ab,kw OR (oligodendroglioma):ti,ab,kw OR (oligodendrogliomas):ti,ab,kw OR (mixed oligodendroglioma-astrocytoma):ti,ab,kw OR (mixed oligodendroglioma astrocytoma):ti,ab,kw OR (mixed oligodendroglioma-astrocytomas):ti,ab,kw OR (anaplastic oligodendroglioma):ti,ab,kw OR (anaplastic oligodendrogliomas):ti,ab,kw OR (oligodendroblastoma):ti,ab,kw OR (oligodendroblastomas):ti,ab,kw
	#3	(Seizure):ti,ab,kw OR (Complex Partial Seizures):ti,ab,kw OR (Complex Partial Seizure):ti,ab,kw OR (Partial Seizure, Complex):ti,ab,kw OR (Partial Seizures, Complex):ti,ab,kw OR (Seizure, Complex Partial):ti,ab,kw OR (Tonic-Clonic Seizures):ti,ab,kw OR (Seizure, Tonic-Clonic):ti,ab,kw OR (Tonic Clonic Seizures):ti,ab,kw OR (Tonic-Clonic Seizure):ti,ab,kw OR (Seizures, Tonic-Clonic):ti,ab,kw OR (Clonic Seizures):ti,ab,kw OR (Seizures, Clonic):ti,ab,kw OR (Epilepsies):ti,ab,kw OR (Epilepsies):ti,ab,kw OR (Seizure Disorder):ti,ab,kw OR (Seizure Disorders):ti,ab,kw OR (Awakening Epilepsy):ti,ab,kw OR (Epilepsy, Awakening):ti,ab,kw OR (Epilepsy, Cryptogenic):ti,ab,kw OR (Cryptogenic Epilepsies):ti,ab,kw OR (Cryptogenic Epilepsy):ti,ab,kw OR (Epilepsies, Cryptogenic):ti,ab,kw OR (Aura):ti,ab,kw

### Selection Criteria

The inclusion criteria were as follows: (1) observational studies and (2) publications in Chinese and English. The exclusion criteria were the following: (1) repeated publications, (2) studies with no full text, incomplete information, or inability to extract data, (3) a different definition of exposure from that encountered in most publications, (4) animal experiments, and (5) case reports, reviews, comments, and systematic reviews.

### Data Extraction and Management

Literature retrieval, screening, and information extraction were independently performed by two researchers. In case of disagreement, a consensus was reached after a discussion with a third researcher. The extracted data included the following: author, year, research type, sample size, and clinical index.

### Quality Assessment

The Newcastle–Ottawa Scale (NOS, http://www.ohri.ca/programs/clinical_epidemiology/oxford.htm) was used by the two researchers to independently evaluate the quality of the included studies. Any disagreement was resolved through a discussion with a third researcher. The NOS includes four items of “research object selection” (four points), one item of “inter-group comparability” (two points), and three items of “outcome measurement” (three points), with a maximum score of nine points; scores ≥7 and <7 points indicate high- and low-quality literature, respectively.

### Data Analysis

Meta-analysis was performed using the R language (version 4.0). Odds ratio (OR) and 95% confidence interval (CI) were used as an effect index to evaluate the correlation between TMMs and the incidence of perioperative epilepsy in patients with glioma. *I*^2^ was used to evaluate heterogeneity. Values of *p* ≥ 0.1 and *I*^2^ ≤ 50% indicated that the studies were homogeneous, and the fixed effects model was used for a combined analysis. Furthermore, *p* < 0.1 and *I*^2^ > 50% indicated study heterogeneity, and sensitivity analysis or subgroup analysis was used to determine the source of heterogeneity. If the heterogeneity remained large, the random effects model was utilized or a descriptive analysis was performed. When a single outcome index included more than 10 articles, funnel plot and Egger's bias test were used to analyze the publication bias of each index.

## Results

### Study Selection

**Figure 1:**
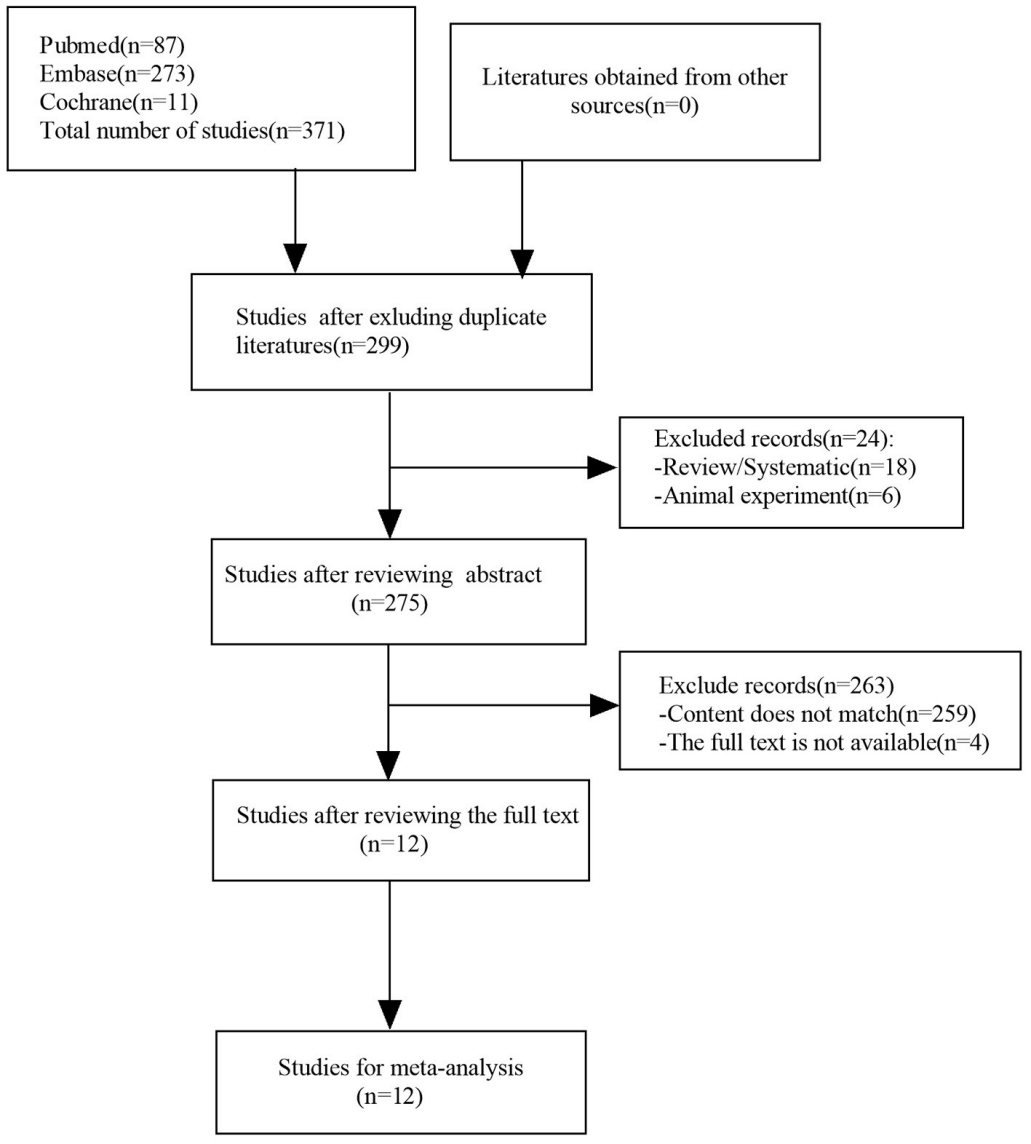
PRISMA flow chart of the included studies.

The comprehensive literature search identified 87 references from PubMed, 273 references from EMBASE, and 11 references from Cochrane (total of 371 references). After removing duplicates, 299 abstracts were reviewed and screened. Twelve studies were selected for full-text evaluation and met the inclusion criteria. Figure 1 illustrates the flow chart.

### Study Characteristics and Quality of Evidence

#### Study Characteristics

The 12 selected cohort studies were published between 2012 and 2020 and conducted in China (*n* = 5), Germany (*n* = 2), the United States of America (*n* = 2), the United Kingdom (*n* = 2), and Australia (*n* = 1). One and 11 studies were prospective and retrospective cohort studies, respectively. The characteristics of the included studies are shown in Table 2.

**Table 2:** Characteristics of the included studies.

**Author**	**Year**	**Study type**	**Study area**	**Sample size**, ***n***		**Sex**, ***n*****(male/female)**		**Age, years**	
				**Seizure**	**Seizure-free**	**Seizure**	**Seizure-free**	**Seizure**	**Seizure-free**
Choi et al.	2020	Retrospective	USA	98	318	59/39	185/133	49.0 ± 14.2	51.1 ± 16.0
Feyissa et al.	2019	Cohort	USA	46	22	28/18	17/5	50 ± 11
Duan et al.	2018	Retrospective	China	73	216	45/28	120/96	48.7	48.7
Neal et al.	2018	Retrospective	China	52	48	/	/	/	/
Roberts et al.	2018	Retrospective	UK	45	18	43/20	34
Yang et al.	2016	Retrospective	China	41	23	/	/	38 (17–72)
Skardelly et al.	2015	Retrospective	Germany	116	218	43/73	88/130	/	/
Zhong et al.	2015	Retrospective	China	221	90	135/86	48/42	37	40.2
Mulligan et al.	2014	Retrospective	UK	48	12	/	/	42 (15–78)
Liubinas et al.	2014	Prospective	Australia	23	7	13/9	2/5	35.4 (17–70)
Liang et al.	2013	Retrospective	China	37	23	34/26	39.5 (17–65)
Stockhammer et al.	2012	Retrospective	Germany	27	22	57	22	40 (13–72)

#### Quality of Evidence

All included studies had a NOS score ≥7, meeting the quality requirement. The specific results are shown in Table 3.

**Table 3:** Evaluation of the study quality.

**Author**	**Year**	**Selection**	**Comparability**	**Exposure**	**Score**
Choi et al.	2020	☆☆	☆☆	☆☆☆	7
Feyissa et al.	2019	☆☆☆	☆☆	☆☆☆	8
Duan et al.	2018	☆☆	☆☆	☆☆☆	7
Neal et al.	2018	☆☆☆	☆☆	☆☆☆	8
Roberts et al.	2018	☆☆	☆☆	☆☆☆	7
Yang et al.	2016	☆☆	☆☆	☆☆☆	7
Skardelly et al.	2015	☆☆☆	☆☆	☆☆☆	8
Zhong et al.	2015	☆☆☆	☆☆	☆☆	7
Mulligan et al.	2014	☆☆☆	☆☆	☆☆☆	8
Liubinas et al.	2014	☆☆	☆☆	☆☆☆	7
Liang et al.	2013	☆☆☆	☆☆	☆☆☆	8
Stockhammer et al.	2012	☆☆☆	☆☆	☆☆☆	8

### Data Analysis

#### IDH Mutation

**Figure 2:**
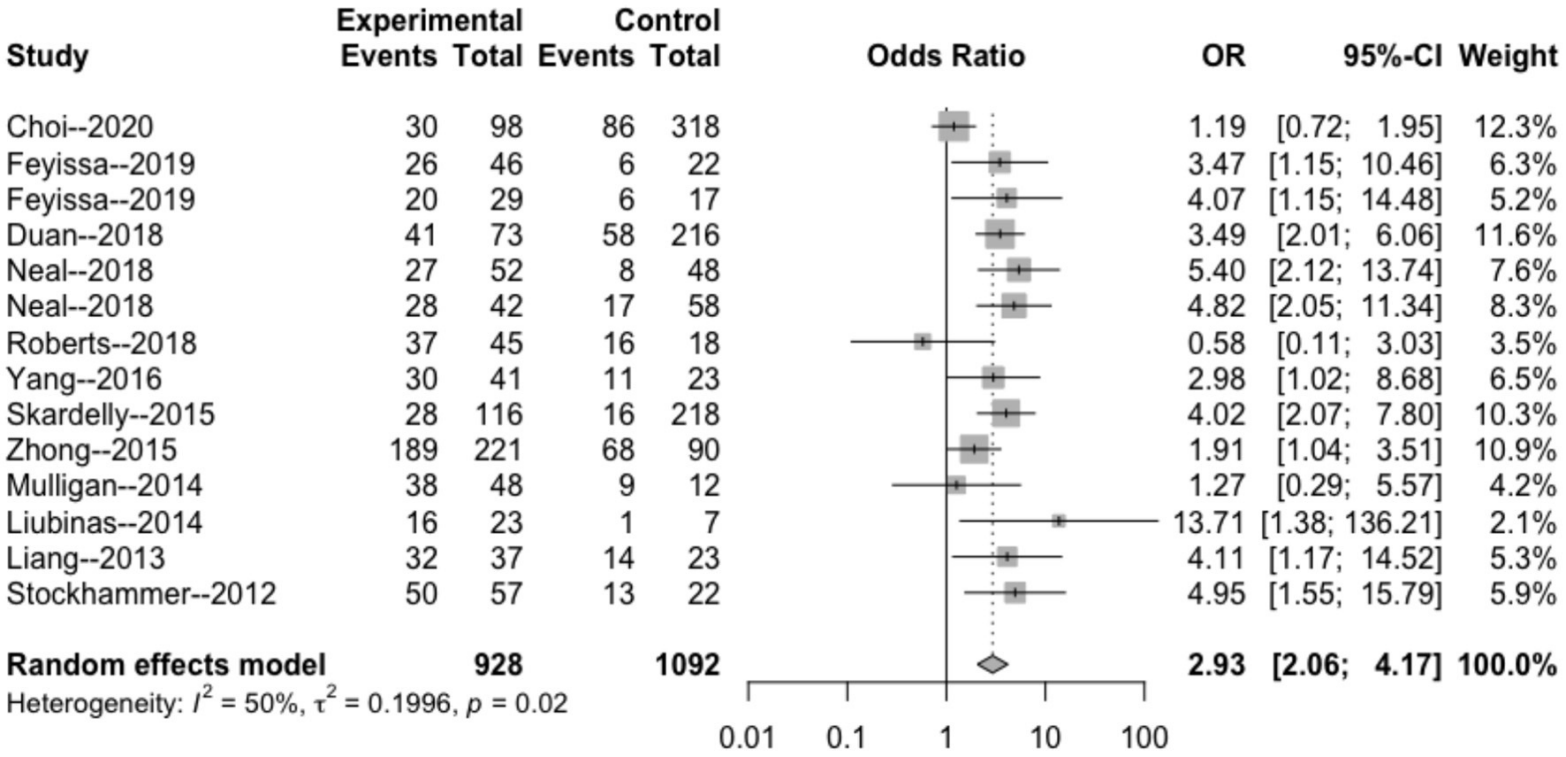
Forest plot of the correlation between IDH mutation and perioperative epilepsy. CI, confidence interval; IDH, isocitrate dehydrogenase; OR, odds ratio.

The heterogeneity testing of 12 studies (*I*^2^ = 50%, *p* = 0.02 < 0.1) showed slight heterogeneity and combined random effects size (OR = 2.93; 95% Cl: 2.06–4.17; *p* = 0.000). These findings suggested a significant correlation between IDH mutation and perioperative epilepsy (Figure 2).

#### MGMT Methylation

**Figure 3:**
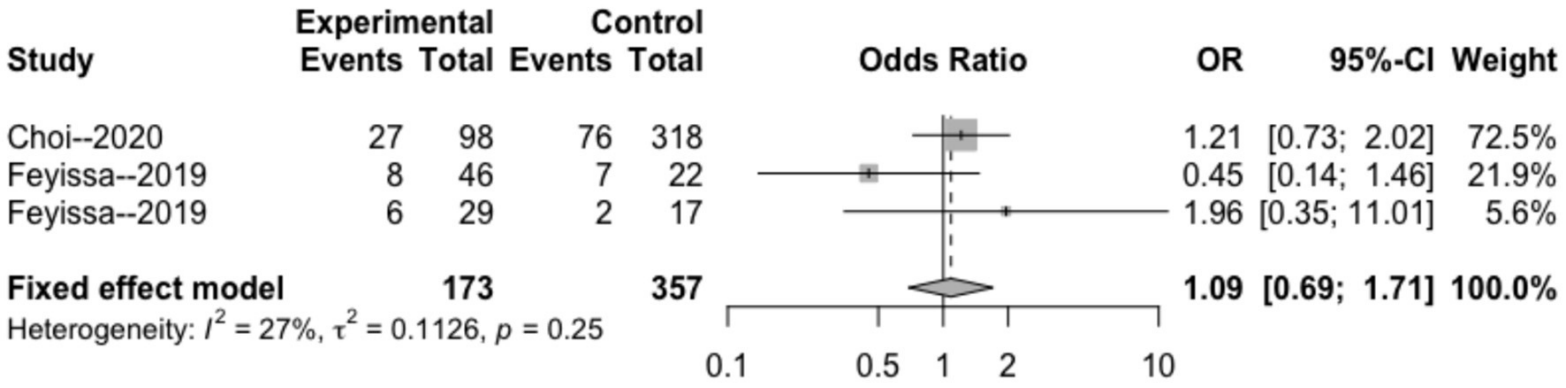
Forest plot of the correlation between MGMT methylation and perioperative epilepsy. CI, confidence interval; MGMT, O6-methylguanine-DNA methyltransferase; OR, odds ratio.

The heterogeneity testing of three studies (*I*^2^ = 27 < 50%, *p* = 0.25 > 0.1) did not show significant heterogeneity in this study and combined fixed effects size (OR = 1.09; 95% Cl: 0.69–1.71; *p* = 0.720). The difference was not statistically significant. There was no correlation between MGMT methylation rate and perioperative epilepsy (Figure 3).

#### 1p/19q Deletion Rate

**Figure 4:**
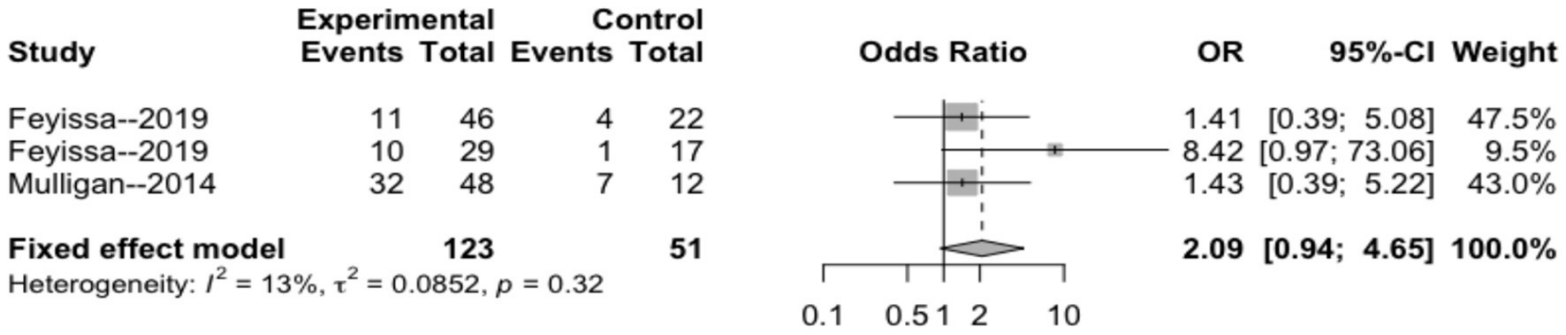
Forest plot of the correlation between 1p/19q deletion and perioperative epilepsy. CI, confidence interval; OR, odds ratio.

The heterogeneity testing of three studies (*I*^2^ = 13 < 50%, *p* = 0.32 > 0.1) did not show significant heterogeneity and combined fixed effect size (OR = 1.40; 95% Cl: 0.94–4.65; *p* = 0.087). The difference was not statistically significant. There was no significant correlation between 1p/19q deletion and perioperative epilepsy (Figure 4).

#### IDH Mutation Subgroup Analysis

**Figure 5:**
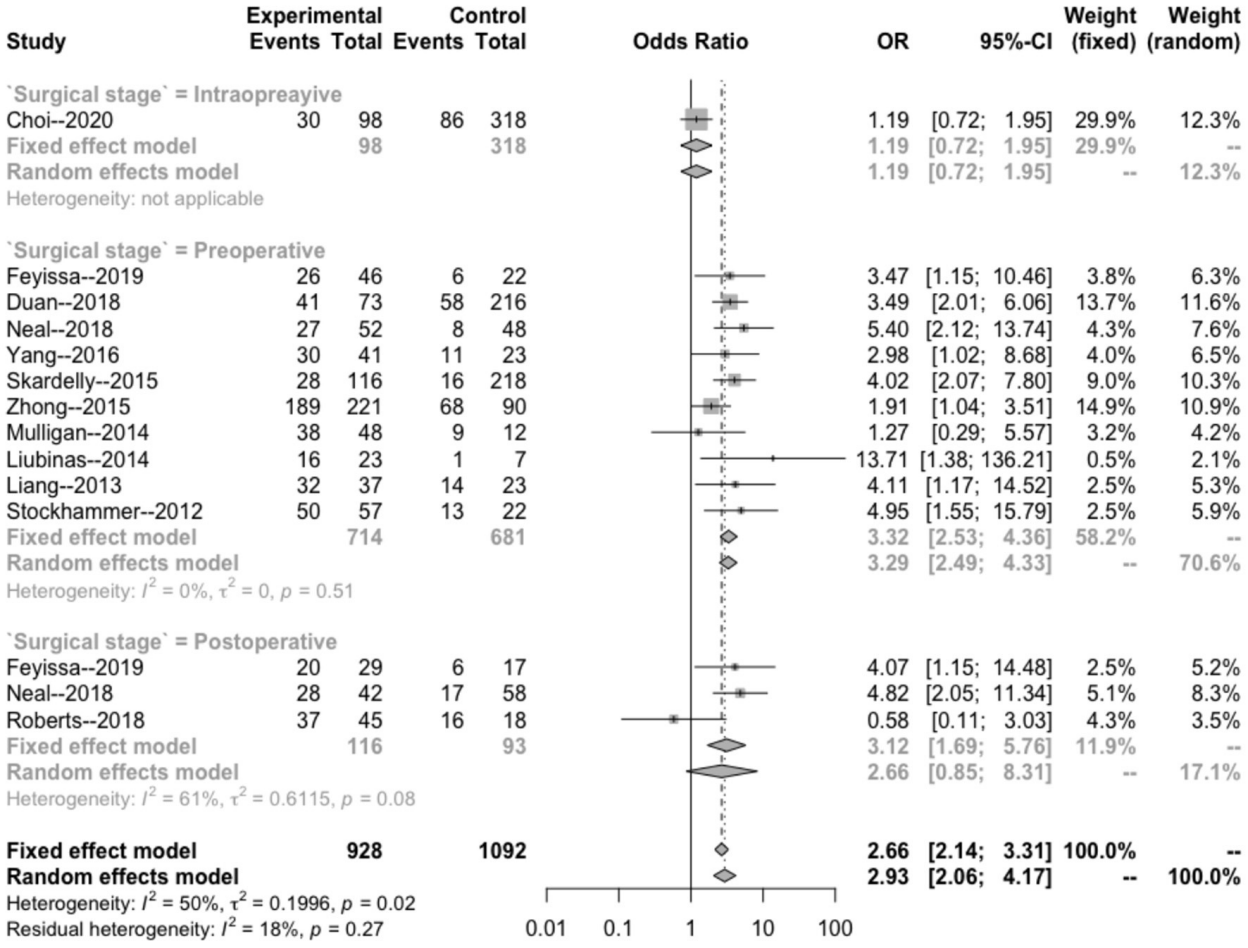
Subgroup analysis of the relationship between IDH mutation and perioperative epilepsy. CI, confidence interval; IDH, isocitrate dehydrogenase; OR, odds ratio.

The intraoperative combination combined effect (OR = 1.19; 95% Cl: 0.72–1.95) did not suggest a significant correlation between the occurrence of intraoperative epilepsy and the presence of IDH in patients with glioma (Figure 5). The heterogeneity within the preoperative group (*I*^2^ = 0 < 50%, *p* = 0.51 > 0.1) and combined effects size (OR = 3.32; 95% Cl: 2.53–4.36) indicated a significant correlation between the occurrence of preoperative epilepsy and IDH in patients with glioma. The heterogeneity within the postoperative group (*I*^2^ = 61 > 50%; *p* = 0.08 < 0.1) and combined effect size (OR = 2.66; 95% Cl: 0.85–8.31) indicated that the occurrence of postoperative epilepsy in patients with glioma was not related to IDH.

#### Bias Test

**Figure 6:**
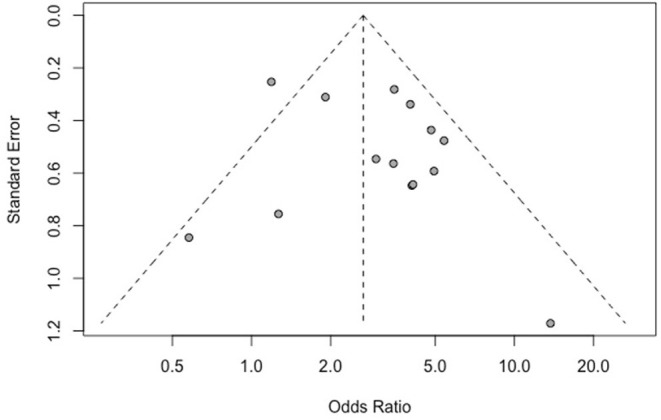
Funnel plot of the correlation between IDH mutation and perioperative epilepsy. IDH, isocitrate dehydrogenase.

The results of Egger's bias test based on the funnel chart of this study (*p* = 0.259 > 0.05) did not indicate an obvious publication bias (Figure 6).

#### Sensitivity Analysis

**Figure 7:**
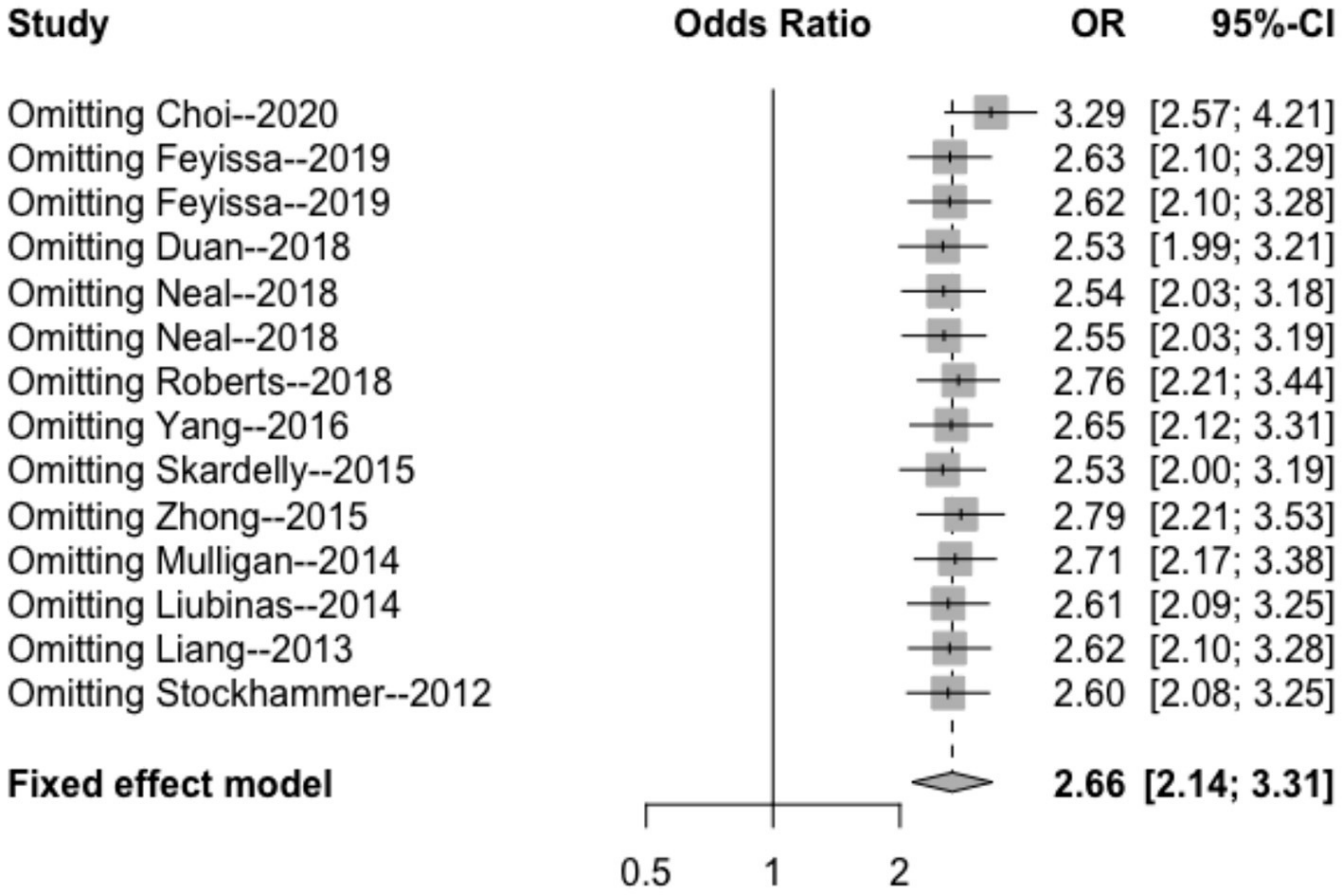
Sensitivity analysis of the correlation between IDH mutation and perioperative epilepsy. CI, confidence interval; IDH, isocitrate dehydrogenase; OR, odds ratio.

**Figure 8:**
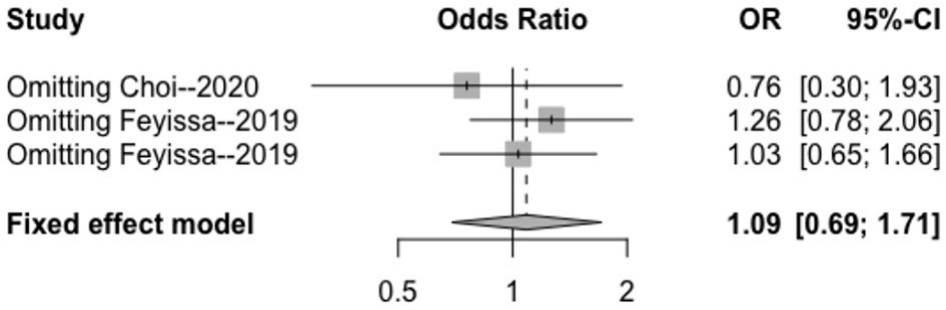
Sensitivity analysis of the correlation between MGMT methylation and perioperative epilepsy. CI, confidence interval; MGMT, O6-methylguanine-DNA methyltransferase; OR, odds ratio.

**Figure 9:**
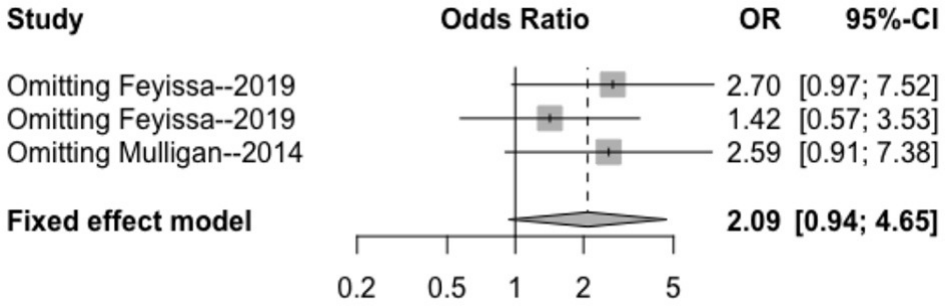
Sensitivity analysis of the correlation between 1p/19q deletion and perioperative epilepsy. CI, confidence interval; OR, odds ratio.

The results of the sensitivity analysis in this study are shown in Figures 7–9. After removing each article in order, it was shown that the individual studies did not have a marked influence on the results. The analysis indicated that the results of this study are relatively stable.

## Discussion

The present meta-analysis confirmed that IDH1 mutation was significantly associated with the incidence of perioperative epilepsy in patients with glioma. In contrast, MGMT methylation and 1p/19q deletion were not related to the incidence of perioperative epilepsy in patients with glioma. Unfortunately, it was not possible to analyze the role of tumor protein p53 (TP53) or epidermal growth factor receptor due to the lack of relevant research literature. The subgroup analysis showed that IDH1 was significantly correlated with the incidence of preoperative epilepsy in patients with glioma; however, it was not significantly correlated with the incidence of intraoperative and postoperative epilepsy. There was no significant heterogeneity and publication bias observed in the included studies, indicating that the results of this meta-analysis are stable.

Previous studies have shown that 1p/19q co-deletion is associated with a better overall survival rate in patients with low-grade glioma. TP53 mutation usually indicates a shorter survival time. IDH1 mutation, MGMT methylation, and 1p/19q co-deletion are good prognostic indicators for low-grade gliomas, being linked to a better overall survival rate (14–16). Interestingly, the combination of IDH1 mutation, MGMT methylation, and TP53 immunopositivity is associated with a faster progression to high-grade tumors (16). Thus far, tumor-related gene changes may be used as a basis for determining the prognosis of glioma. Nevertheless, this tendency has not been well investigated as a potential biomarker of epilepsy. Researchers have been committed to exploring the biochemical changes in the tumor microenvironment, searching for pathogenic and influencing factors of tumor-related epilepsy at the molecular level.

Duan et al. reported that IDH1 mutations are significantly associated with preoperative seizures in patients with glioma (17). Recently, Feyissa et al. confirmed that IDH1 mutation and MGMT methylation are associated with the occurrence of perioperative seizures (10). However, Liubinas et al. found that the presence of IDH1 mutations is not related to the incidence of epilepsy (18), though the results of that study may have been influenced by the inclusion of higher-grade tumors. Yang et al. reported that patients with a low expression of the MGMT protein were more likely to experience seizures after operation (19). In contrast, Feyissa et al. found that methylation of the MGMT gene promoter was associated with increased postoperative seizure control (10). However, they did not investigate other TMMs thought to be related to postoperative seizure control, which may have influenced their findings. Mulligan et al. showed that the combined deletion of 1p/19q was not related to the occurrence of preoperative seizures in patients with low-grade oligodendrogliomas (8). Moreover, due to the limited number of related studies, this meta-analysis could not determine the relationship between TMMs and perioperative seizures in patients with tumor-grade glioma.

The mechanism of tumor-related epilepsy remains unclear. The present findings showed that IDH1 mutations are associated with perioperative epilepsy; the subgroup analysis further showed that IDH1 is significantly related to preoperative epilepsy. The underlying mechanism of IDH mutation and perioperative seizures is unclear. This may be attributed to the activation of the receptor of N-methyl-D-aspartate (NMDA) by D-2-hydroxyglutarate (D2HG), which has a very similar structure to that of glutamate (20). Exogenous D2HG can increase the discharge of neurons in rats. The excitatory effect of D2HG could be blocked by treatment with a selective NMDA receptor antagonist (21). Numerous studies have also shown that the concentration of D2HG in glioma cells carrying IDH1 mutations is 100–300-fold higher than that measured in normal tissues (22). These studies suggested that D2HG may promote the occurrence and development of epilepsy in patients with glioma. Moreover, some studies have shown that perioperative epilepsy is not related to the location, grade, or histopathology of glioma (10, 23, 24). Previous studies have also shown that seizures may not occur in all patients with similar glioma localization and histology (24, 25). Therefore, the heterogeneity of TMM in gliomas may lead to the observed variability of epilepsy in patients with glioma. These results are important for the research and treatment of glioma-related epilepsy.

Although our results did not have any publication bias or statistically significant heterogeneity, the present analysis included 11 retrospective studies and no randomized studies. Moreover, there are still fewer TMMs other than IDH1 mutations. Hence, further studies are warranted to verify the present findings.

## Conclusion

Our findings provide supporting evidence for the correlation between TMMs and glioma-related seizures. Future studies should include epileptic symptoms and detect more TMMs. Clarifying the mechanism of perioperative epilepsy in glioma will benefit the clinical treatment of patients with this disease and the precise treatment of epilepsy.

## Data Availability Statement

The original contributions presented in the study are included in the article/supplementary material, further inquiries can be directed to the corresponding author/s.

## Author Contributions

LS and XQ performed the data collection, wrote and revised the manuscript, and are co-first authors. LC designed the study. JZ contributed to conceiving and revising the manuscript. LC and JZ are co-corresponding authors. CC performed the data collection and analysis. All authors contributed to the article and approved the submitted version.

## Conflict of Interest

The authors declare that the research was conducted in the absence of any commercial or financial relationships that could be construed as a potential conflict of interest.

## Publisher's Note

All claims expressed in this article are solely those of the authors and do not necessarily represent those of their affiliated organizations, or those of the publisher, the editors and the reviewers. Any product that may be evaluated in this article, or claim that may be made by its manufacturer, is not guaranteed or endorsed by the publisher.
